# Photokinetic Drug Delivery: Near infrared (NIR) Induced Permeation Enhancement of Bevacizumab, Ranibizumab and Aflibercept through Human Sclera

**DOI:** 10.1007/s11095-018-2392-7

**Published:** 2018-03-29

**Authors:** Steven A. Giannos, Edward R. Kraft, Zhen-Yang Zhao, Kevin H. Merkley, Jiyang Cai

**Affiliations:** Department of Ophthalmology and Visual Sciences, University of Texas Medical Branch, 301 University Blvd, Galveston, Texas 77555 USA

**Keywords:** aflibercept, bevacizumab, permeation, photokinetic, ranibizumab, sclera

## Abstract

**Purpose:**

Permeation studies, with near infrared (NIR) light and anti-aggregation antibody formulation, were used to investigate the *in vitro* permeation of bevacizumab, ranibizumab and aflibercept through human sclera.

**Methods:**

A vertical, spherical Franz cell diffusion apparatus was used for this scleral tissue permeation model. A photokinetic ocular drug delivery (PODD) testing device accommodated the placement of NIR LEDs above the donor chambers. An adjustable LED driver/square wave generator provided electrical energy with a variable pulse rate and pulse width modulation (duty cycle).

**Results:**

Exposure to non-thermal NIR light had no effect on mAbs with regard to monomer concentration or antibody binding potential, as determined by SE-HPLC and ELISA. The optimal LED wavelength was found to be 950 nm. Duty cycle power of 5% *vs* 20% showed no difference in permeation. When compared to controls, the combination of non-aggregating antibody formulation and NIR illumination provided an average transscleral drug flux enhancement factor of 3X.

**Conclusion:**

Narrow wavelength incoherent (non-laser) light from an NIR LED source is not harmful to mAbs and can be used to enhance drug permeation through scleral tissue. The topical formulation, combined with pulsed NIR light irradiation, significantly improved scleral permeation of three anti-VEGF antibody drugs.

## Introduction

The number of people visually impaired in the world is estimated to be 285 million, 39 million blind and 246 million having low vision; 65% of people visually impaired and 82% of all blind are 50 years and older. (1) Age-related macular degeneration (AMD) is a progressive, degenerative disease of the retina that occurs with increasing incidence with age and ranks third among the global causes of visual impairment. (1) Exudative AMD is caused by new, abnormal blood vessel growth (neovascularization) in the subretinal layers, leading to vascular leaks, bleeding, and progressive vision loss. (2) Vascular endothelial growth factor (VEGF) is a signaling protein produced by macrophages, retinal pigment epithelium and Muller cells that stimulate vasculogenesis and angiogenesis. Overexpression of VEGF has been implicated in the development and progression of neovascular AMD. (3) Drugs such as the monoclonal antibodies, bevacizumab, ranibizumab and the fusion protein, aflibercept can inhibit VEGF and control or slow those diseases. (4–6)

The commercial development of therapeutic monoclonal antibodies commenced in the early 1980’s, and by 1986 the first therapeutic monoclonal antibody (mAb), Orthoclone OKT3, was approved for the prevention of kidney transplant rejection. (7) As of 2015, the highly dynamic late-stage commercial pipeline of recombinant therapeutics now includes nearly 50 molecules. (8) The majority of approved antibody drugs are used to treat cancer and inflammation. However, two of these monoclonal antibodies, bevacizumab (Avastin®) and FDA approved ranibizumab (Lucentis®), show anti-VEGF properties and may be used to treat age related macular degeneration (AMD) and diabetic retinopathy. (9)

VEGF-Trap (Aflibercept, Intravitreal aflibercept injection (IAI Eylea®)) is a soluble VEGF decoy receptor that consists of the second immunoglobulin (Ig)-like domain of FLT1 and the third Ig-like domain of KDR (kinase insert domain receptor) linked to the IgG constant region (Fc). (10) Eylea®, the FDA approved formulation of aflibercept for the treatment of wet AMD, is administered as an intravitreal injection. (11)

Systemic drug administration for ophthalmic disease is difficult because of poor drug permeability, due to a blood-retinal barrier, encountered when targeting the posterior segment. (12,13) The retina has a unique position with regard to pharmacokinetics in that the blood-retinal barrier (BRB) separates the retina from the circulating blood. The BRB, which forms complex tight junctions of retinal capillary endothelial cells (inner BRB) and retinal pigment epithelial cells (outer BRB), restricts nonspecific transport between the neural retina and the circulating blood. (14)

Anti-VEGF drugs are delivered by repeated intravitreal injections (15), which can bring about serious complications including retinal detachment and infection, as well as being painful and costly. (16) Alternative approaches such as iontophoresis (17), sonophoresis (18) and photokinetic drug delivery (19) have been used, non-invasively, to deliver drugs to the back of the eye.

Photokinetic drug delivery, for transdermal and ocular applications, is a new addition to noninvasive drug delivery therapies. (20) Photokinetic facilitated drug delivery uses selected pulsed illumination with a selected light wavelength, directed onto drug molecules residing on a tissue surface. The selected cyclic illumination causes unidirectional translocation of the drug molecules from the tissue surface into the tissue. Godley *et al*. recently described their early work with photokinetics and the development of a device to deliver light energy, especially in the application of transscleral drug delivery, to the posterior segment of the eye. (19) This strategy was applied and investigated as a simplified model of scleral-vitreous interface and unstirred gel mimicking the vitreous. (21)

The focus of this research is to show the feasibility of transscleral mAbs permeation, as well as enhanced permeation due to NIR light irradiation. While developing and validating methods for the photokinetic enhancement of transscleral mAb permeation, using an *in vitro* Franz diffusion model, we encountered the problem of mAb dilute solution instability in phosphate buffered saline (PBS). Published tissue permeation (Franz) cell studies typically use PBS as the recipient media at 37°C. Franz cell studies start with a recipient media concentration of 0 of the test compound. We have identified a phenomenon wherein low concentrations of antibodies (<1150 ng/ml), in PBS or the manufacturer’s formula, lose monomer concentration.

As the permeation experiment progresses; the concentration of the test compound increases. However, at early points in the tissue permeation experiment, the drug is still a very dilute solution (i.e., at 2 h, it could be anywhere between 0 and 5000 ng/ml). For our experimental work on anti-VEGF mAbs, we found that these conditions contributed to mAb aggregation, causing reduced monomer concentrations and decreased VEGF binding capacity.

Scleral drug permeation of large entities is generally molecular weight and molecular size limited. (22) Antibody aggregation, combining two or more individual antibodies, would have a significant permeation rate limiting effect. Doubling or tripling of a topically applied drug weight and size by aggregation would render these large drug structures impossible to permeate sclera. Antibody aggregation within the topically applied drug composition had to be eliminated before any assessment of passive and facilitated permeation could be evaluated.

We have developed and used a novel, stabilized formulation that prevents the antibodies from aggregating, which is suitable for topical ocular delivery. (23,24) *In vitro* studies demonstrate that this formulation, with concurrent non-thermal NIR light irradiation, delivers clinically relevant drug amounts, noninvasively, through scleral tissue using a one-hour treatment.

## Materials and Methods

### Materials

L-Argininine, sodium phosphate dibasic anhydrous, sodium phosphate monobasic monohydrate, sodium sulfate anhydrous, phosphate buffered saline (10X), and HPLC water were obtained from Fisher Scientific Fair Lawn, NJ. α,α-trehalose dihydrate, polysorbate 80 (Tween® 80, low peroxide), and sodium chloride, USP was purchased from Sigma-Aldrich, St Louis Mo and normal saline 0.9% obtained from Baxter Healthcare Corp., Deerfield, IL.

ELISA analytical kits for bevacizumab (kit #AVA-E-U51) and Ranibizumab (kit #LUC-E-U52) were obtained from United Immunoassay Inc., San Bruno, CA USA. Aflibercept was analyzed using an ELISA procedure as described Celik *et al*. (25)

Antibodies bevacizumab (Avastin®) 25 mg/ml and ranibizumab (Lucentis®) 10 mg/ml were obtained from Genentech, South San Francisco CA. Intravitreal aflibercept injection (IAI Eylea®) 48.2 mg/ml was obtained from Regeneron, Tarrytown NY. (Note: bulk aflibercept as provided by Regeneron is at 48.2 mg/ml while the common pharmaceutical preparation Eylea® is formulated at 40.0 mg/ml). All other reagents were of analytical or USP purity. Whole globe human donor eye pairs, designated for research, were obtained from Lone Star Lions Eye Bank, Manor, TX, from Lions Eye Bank of Texas/ Baylor College of Medicine, Houston Texas and from TBI Orlando/ Medical Eye Bank of Florida.

### Light Device and Experimental Setup

### Photokinetic/Diffusion Apparatus

**Fig. 1:**
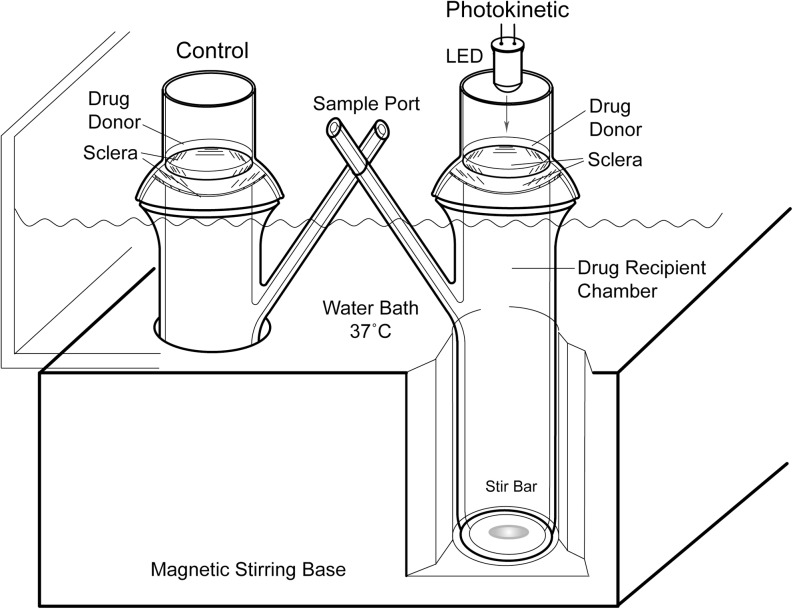
Schematic of Franz cell diffusion apparatus.

A vertical, spherical Franz cell diffusion apparatus was adapted and used for the sclera tissue permeation model. Spherical Franz cells (PermeGear, Inc., Hellertown, PA) having a 9.1 mm diameter (0.65 cm^2^ diffusional area) were used. A photokinetic ocular drug delivery (PODD)-modified Franz cell testing device was configured so that it accommodated the placement of NIR LEDs within the donor chamber. The LEDs are placed 1 cm above the surface of the scleral membrane. The cells were placed within an aluminum block on a magnetic stir bar setup (manufactured by PermeGear, Inc., Hellertown, PA), with a custom built 37°C water bath to provide even temperature control within the aluminum block, the water bath and the permeation cells. The experimental arrangement is shown in Fig. 1.

Spherical Franz cells were adapted for sclera photokinetic permeation studies. A donor cell, containing bevacizumab, ranibizumab or aflibercept in Formula 14 (F14, described below) was on top of the scleral membrane and receiver cell. The receiver cell was filled with F14 as well. Samples for chemical analysis were taken from the side arm of the Franz apparatus with volume replacement. Control cells were set up the same, but without the NIR LED. NIR LEDs were driven by a custom built square wave pulse generator.

An adjustable square wave pulse generator provided pulsed electrical energy from 400 to 1000 cycles per second (CPS) with a variable pulse width modulation duty cycle circuit. This circuit is adjustable from 0.1% to 99% percent ON pulse duration (e.g., ON 50%, OFF 50% of the time, 50% duty cycle; or ON 10%, OFF 90%, 10% duty cycle; or ON 20%, OFF 80%, 20% duty cycle and so on). The electrical current for the driver of the LEDs was adjusted to about 5 to 8 times above the continuous wave current level recommended for the LED drive current in order to provide the desired equivalent rated optical power output with short duration pulsed drive current. In general, the short duration electrical pulse (i.e. 10–20% duty cycle) with the over-rated drive current, provided a minor (≤ 1°C) donor compartment temperature increase above ambient conditions. Combination adjustments in pulse widths in conjunction with electrical drive currents were used to produce the required optical power without excess or extraneous LED device heat or heat from absorbed light energy. The temperature of the drug donor compartment was ≤1°C above ambient Franz cell apparatus temperature.

### Drug Permeation Experiments

### Drug Formulation

Bevacizumab, ranibizumab or aflibercept was placed in Formulation 14 (F14), which contains 100 mM sodium phosphate buffer, 0.3% NaCl, 7.5% trehalose, 10 mM arginine and 0.04% Tween 80 at a pH of 6.78. (23) The composition was used as a diluent for the drug donor solution and also used as the drug recipient media in the permeation cell studies. Aflibercept (as provided), ranibizumab and bevacizumab had different packaged concentrations (43.8, 10 and 25 mg/ml respectively), and were diluted to 2.5 mg/ml. A total standardized dosage of 1 mg was provided by placing 0.4 ml of the 2.5 mg/ml solution in the Franz cell. The antibody solution was left in place for one hour then removed from the donor chamber.

### Tissue Protocol

Whole globe human donor eye pairs, designated for research, were obtained from Lone Star Lions Eye Bank, Manor, TX, from Lions Eye Bank of Texas/ Baylor College of Medicine, Houston Texas and from TBI Orlando/ Medical Eye Bank of Florida. Eye pairs were positioned in moist chambers with transport cages and shipped on wet ice or otherwise stored at 4°C. The average time of death to time of permeation study start was 84.48 h, ranging from 39 to 171 h. The eyes were dissected into 4 sections from anterior to posterior along the axis of the muscle insertion points to assure that blood vessel penetrations would not be within the permeation area and be positioned under the Franz cell flange. Anterior tissue sections from limbus to equator were mounted onto 0.65cm^2^ hemispheric Franz cells and clamped into place. Cells were checked to make sure that there was no leakage through the tissue between the donor and receiver chambers and also no leakage from the donor or receiver chambers to the exterior of the Franz cell. Each group of Franz cells, the 4 sections from each eye, were randomly designated to either a control or a photokinetic group; i.e. right eye verses left eye. Results were multiplied by 1.54 to provide amount of drug permeated per 1cm^2^ for convenience and comparison, as is generally accepted.

### Experimental Protocol

After temperature equilibration, the donor chamber was emptied and dried. The test drug in formula 14, 400ul of 2.5 mg/ml (1 mg total), was added into the donor chambers. The control group was held in the dark. The photokinetic group had a selected NIR LED positioned 1 cm above the tissue surface. Experimental time started when the drug solution was placed in the donor chamber and NIR LED turned ON together. The drug solution, with the concurrent NIR irradiation, was allowed to sit for 60 min. After 60 min, the drug solution was removed from the donor chamber and the NIR LED was turned OFF. In our studies, we did not vary the length of time for the application of the antibody solution or the length of time of NIR irradiation. These parameters were held constant at 60 min.

Serial 400ul samples were taken from the center of the recipient chamber with a 3″ x 20G needle and syringe at the selected time points. Sample volume was replaced with degassed F14. Samples were centrifuged at 1090 relative centrifugal force (RCF) for 5 min, at 23°C, to precipitate any tissue debris.

### Sample Analysis

### Enzyme-Linked Immunosorbent Assay (ELISA)

Bevacizumab and ranibizumab ELISA assays were performed as per manufacturer’s instructions except for a substitution of the base analytical standard material which was taken from the pharmaceutical preparations obtained and diluted as described below. Aflibercept ELISA was performed as described by Celik *et al*. (25), except for a substitution of the base analytical standard material which was taken from the pharmaceutical preparations obtained and diluted as described below. The ELISA method dynamic ranges are as follows: bevacizumab 1–281.25 ng/ml, ranibizumab 1-125 ng/ml and aflibercept 1-125 ng/ml.

### Size-Exclusion High Performance Liquid Chromatography (SE-HPLC)

Analytical size-exclusion chromatography was performed using an Agilent HPLC system HP1100 from Agilent Technologies (Santa Clara, CA) with a UV detector. Studies were performed using a TSKgel UltraSW Aggregate 7.8 mm × 30 cm, 3 μm SEC column with TSKgel UltraSW guard column (Tosoh Bioscience LLC, King of Prussia, PA). Mobile phase comprising 85% 100 mM sodium sulfate in 100 mM phosphate buffer in HPLC water (adjusted to pH 6.68) with 15% acetonitrile/ 0.1% trifluoroacetic acid was used at a flow rate of 0.6 ml/min. Sample injections were 100 μl in volume. The eluted protein was monitored by UV Absorbance at 212 nm. Silanized HPLC sample vials and silanized vial inserts from Agilent Technologies (Santa Clara, CA), were used throughout.

### Statistical Analysis

Data is presented as mean ± Standard Deviation (SD). Group to group analysis was conducted with student’s two-tailed t-test using Microsoft Excel. In all cases, a *p* value ≤0.05 is considered to be significant.

### ***In Vitro*** Studies

### **Bevacizumab/NIR Light Stability Study**

It is well known that ultraviolet light may degrade an antibody. However, we were interested in finding if non-thermal, non-ionizing infrared light had any detrimental effects on antibodies. In this case a volume of 25 mg/ml bevacizumab was diluted with F14 to a concentration of 500 ng/ml. This material was used as a starting composition and separated into two groups; control and light exposure, n = 4/group. Plastic chambers (3 ml) were fitted with tight lids, housing 5 mm 950 nm NIR LEDs with light directed toward the bottom of the chambers. Each chamber received 600 μl of the 500 ng/ml bevacizumab in F14. LEDs were positioned 3 cm above the surface of the antibody solution. The chambers were placed in a 37°C water bath. In the light control group, the LED was turned ON providing 24.5 ± 2.5 mW power, fluency of 3.15 ± 0.15 W/cm^2^ pulsed at 1000 cycles per second (Hz) with a duty cycle of 20% (20% ON time) measured at the LED surface. At 3 cm distance from the LED to the surface of the solution, the power was 12 mW ± 1.0 mW and a fluency of 1.25 W ± 0.25. The non-light control group was shielded from light exposure. Samples were taken at 1 h and 5 h and examined by ELISA and SE-HPLC.

A standard dilution range of 1125 to 4.3945 ng/ml starting with 25 mg/ml bevacizumab in F14 was made and divided for both analytical methods. ELISA standard dilutions and test samples were run on the same plate at the same time. SE-HPLC standard dilution and test samples were run consecutively on the same method setup. Standard dilution curves were derived and used to determine the concentration of the subject test samples for each method.

### Photokinetic/Bevacizumab Antibody Permeation - Different IR Wavelengths

In this study, 5 different NIR wavelengths were tested, with bevacizumab in F14, to identify the best condition for photokinetic drug delivery. Human sclera was used as the test membrane. The drug donor was 400ul of 2.5 mg/ml bevacizumab in each Franz cell, which was removed at one hour and the LED was turned off. Samples were taken at 5 and 8 h. The conditions tested were: 830 nm/1000cps/5% duty cycle (ON time 5% *vs* OFF time 95%), 950 nm/1000cps/5% duty cycle, 1300 nm/1000cps/5% duty cycle, 1450 nm/1000cps/5% duty cycle, 3400 nm/400cps/2.5% duty cycle. There was no heat generated from the LEDs at these duty cycles. All samples were analyzed by ELISA.

### ELISA ***vs***. SE-HPLC

An *in vitro* study, using ranibizumab, was run to test the correlation of ELISA results to SE-HPLC analysis results using F14 and NIR light. In this study, the NIR irradiation was applied for 1 h; dark control *vs* 950 nm/1000cps/20% duty cycle (27mw/cm^2^). The controls were shielded from light. For this experiment, a single eye donor, with 4 sclera sections per eye, was used. Right eye *vs*. left eye was tested. The drug donor was 400ul of 2.5 mg/ml bevacizumab in each Franz cell and was removed at one hour and LED was turned off. Samples of 400ul were taken at 2, 3, 5, 8, 10, 12 and 24 h.

SE-HPLC was used to accurately quantitate the monomeric antibody concentration over a wide dynamic range. SE-HPLC, however, is not an indicator of biological activity. ELISA, which is an additional method to determine concentration, has a generally limited dynamic range. ELISA is not a robust quantitative method, but it clearly demonstrates the biological activity of the antibody. Together, quantification was determined and correlated using physico-chemical and biological activity methods.

The samples were split in two for SE-HPLC and ELISA analysis. SE-HPLC analysis was started immediately. Derived SE-HPLC values determined the dilution strategy of samples for ELISA to bring into the usable range of the method. The ELISA range was 1-200 ng/ml. The SE-HPLC range was 4.4–18,000 ng/ml. ELISA samples were diluted 2X -25X as determined by SE-HPLC results. ELISA was run for the 3, 5, 8, 12 & 24 h time points (samples from 2 h and 10 h time points were omitted due to limited space on the ELISA test plates).

### Formula 14/Bevacizumab Sclera Permeation Feasibility Study

An initial feasibility *in vitro* human sclera permeation study was performed, with bevacizumab, to replicate F14 exposure to NIR light (n= 18). In this study, NIR irradiation of 950 nm/1000cps/5% duty cycle (8mw/cm^2^) was used. The controls were shielded from light and the NIR irradiation was applied for 1 h. After this time point, the NIR light was turned off and the drug solution was removed. Samples were taken at 5, and 8 h.

### Same Wavelength (950 nm), Different Duty Cycle (5% ***vs***. 20%)

In this study, the same wavelength LED (950 nm) was tested, with bevacizumab at two different duty cycles, in order to study the impact of power level on photokinetic drug delivery. Human sclera was used as the test membrane. The drug donor was 400ul of 2.5 mg/ml bevacizumab in each Franz cell and was removed at one hour and LED was turned off. Samples (400 μl) were taken at 2, 3, 5, 8, 10 and 12 h. The conditions tested were: dark control *vs* 950 nm/1000cps/5% duty cycle (8mw/cm^2^) and dark control *vs* 950 nm/1000cps/20% duty cycle (27mw/cm^2^). Samples were analyzed by SE-HPLC.

### Ranibizumab with NIR 950 nm

Ranibizumab was diluted to 2.5 mg/ml and then 400ul (1 mg) was placed in each Franz cell (*n* = 16). The photokinetic cells were irradiated with 950 nm light, 1000cps, 20% duty cycle (27 mW/ cm^2^) for the exposure hour. The donor drug solution removed at one hour. Control cells were held in the dark for the exposure hour. Samples (400 μl) were taken at 2, 3, 5, 8, 10, 12 and 24 h.

### Aflibercept with NIR 950 nm

Aflibercept was diluted to 2.5 mg/ml and then 400ul (1 mg) was placed in each Franz cell. The photokinetic cells were irradiated with 950 nm light, 1000cps, 20% duty cycle (27 mW/ cm^2^) for the exposure hour. The donor drug solution was removed at one hour. Control cells were held in the dark for the exposure hour. Samples (400 μl) were taken at 2, 3, 5, 8, 10, 12 and 24 h.

## Results

### ***In Vitro*** Studies

### Bevacizumab/NIR Light Stability Study

**Fig. 2:**
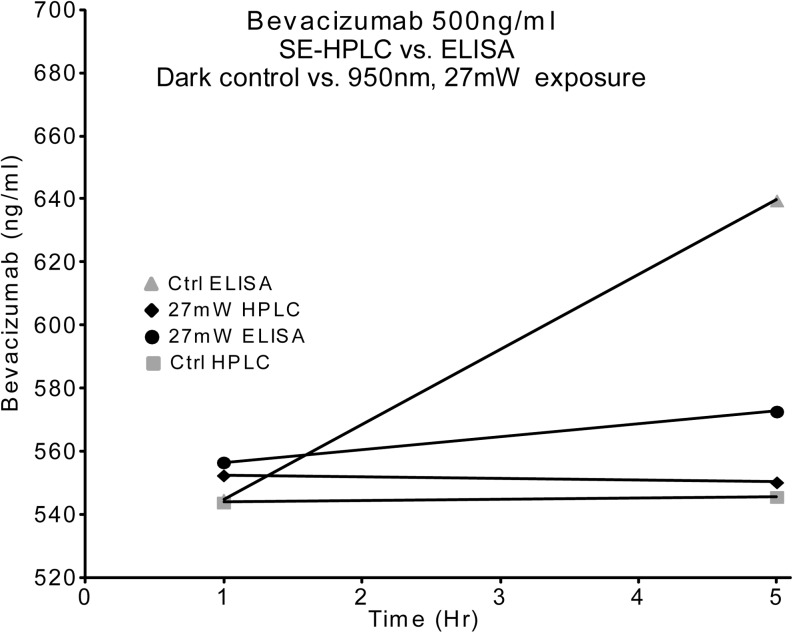
Graphical comparison of analytical methods for bevacizumab exposed to 950 nm IR light from 1 to 5 h. Samples run in duplicate for each of the 4 conditions ( n= 2).

Figure 2 shows the result of NIR light exposure using both ELISA and SE-HPLC methods for analysis. The bevacizumab concentrations obtained for the control (no NIR light) and light exposed (NIR light) groups, as analyzed by SE-HPLC, were both about 550 ng/ml at 1 and 5 h, respectively. The bevacizumab concentrations obtained for the one hour control and light exposed group were also about 550 ng/ml, using ELISA as the analytical method. At 5 h, both the control and the light exposed groups showed a slight increase in concentration, when analyzed by ELISA. This slight increase in concentration may be attributed to the inherent error in ELISA methodology and/or standard curve dilution or interpolation errors. In any event, it appears that exposure to relatively high amounts of non-thermal NIR light does not have a detrimental effect on bevacizumab diluted with formula 14.

### Different NIR Wavelengths

**Fig. 3:**
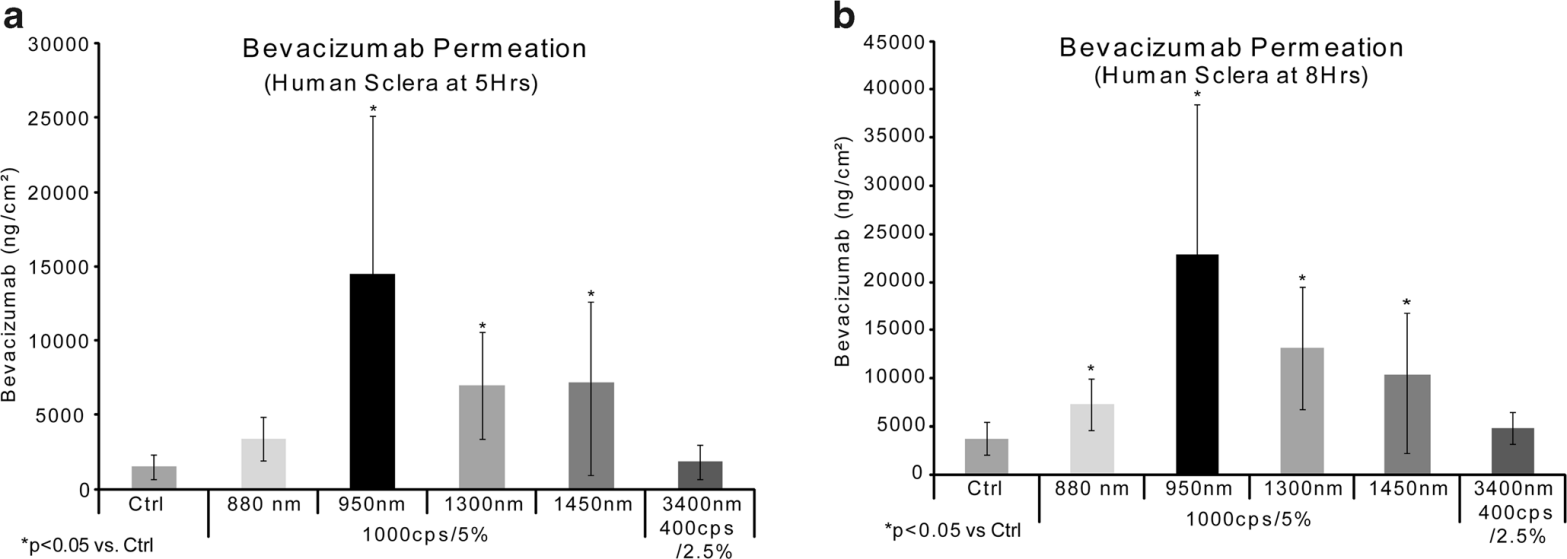
Results from the wavelength study, control *vs* light conditions. For Fig. 3a and b: control  n= 8, 880  n= 4, 950 n = 8, 1300 n = 4, 1450 n = 4 and 3400 n = 4.

Table I shows the results of a study of comparing bevacizumab and NIR irradiation using different wavelengths. Samples were taken at 5 and 8 h. As can be seen in Fig. 3, the best results were obtained from the 950 nm wavelength, when comparing 880, 950, 1300, 1450 and 3400 nm IR light.

**Table I:** Scleral permeation of bevacizumab using different NIR wavelengths

Freq (nm)	CPS	Duty Cycle	5 Hrs. ng/ml (SD)	8 Hrs. ng/ml (SD)
Control	1000	5%	1503 (814)	3749 (1671)
880	1000	5%	3378 (1416)*	7335(2659)*
950	1000	5%	14,500 (10630)*	22,888 (15543)*
1300	1000	5%	7009 (3588)*	13,101 (6358)*
1450	1000	5%	7206 (7371)	10,325 (9302)
3400	400	2.50%	1836 (1156)	4816 (1625)

*p < 0.05

### Formula 14/Bevacizumab Sclera Permeation Feasibility Study

**Fig. 4:**
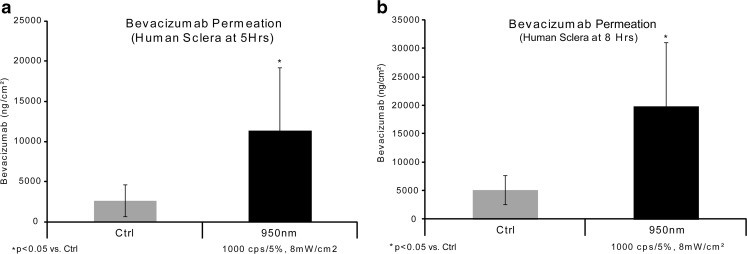
Feasibility testing of Formula 14. A 950 nm/1000cps/5% duty cycle appears to provide a reliable 4X enhancement of permeation. For Fig. 4a and b: control  n= 10 and 950nm n = 10.

Figure 4 shows the results from the feasibility experiments (n= 18/group). The control bevacizumab permeation (no NIR light) was 2671 ± 1967 ng/cm^2^ at 5 h (Fig. 4a) and 5094 ± 2523 ng/cm^2^ at 8 h (Fig. 4b). The NIR light irradiated samples (950 nm/1000cps/5% duty cycle (8 mW/cm^2^)) were 11,390 ± 7872 ng/cm^2^ at 5 h (Fig. 4a) and 19,859 ± 11,226 ng/cm^2^ at 8 h (Fig. 4b). This test successfully shows that mAbs may be evaluated using this equipment, formula and method. The results also show that using NIR light significantly (*p* < 0.05) increases transscleral drug delivery of bevacizumab, as well as providing a reliable 4X enhancement of permeation.

### Same Wavelength (950 nm), Different Duty Cycle (5% ***vs***. 20%)

**Fig. 5:**
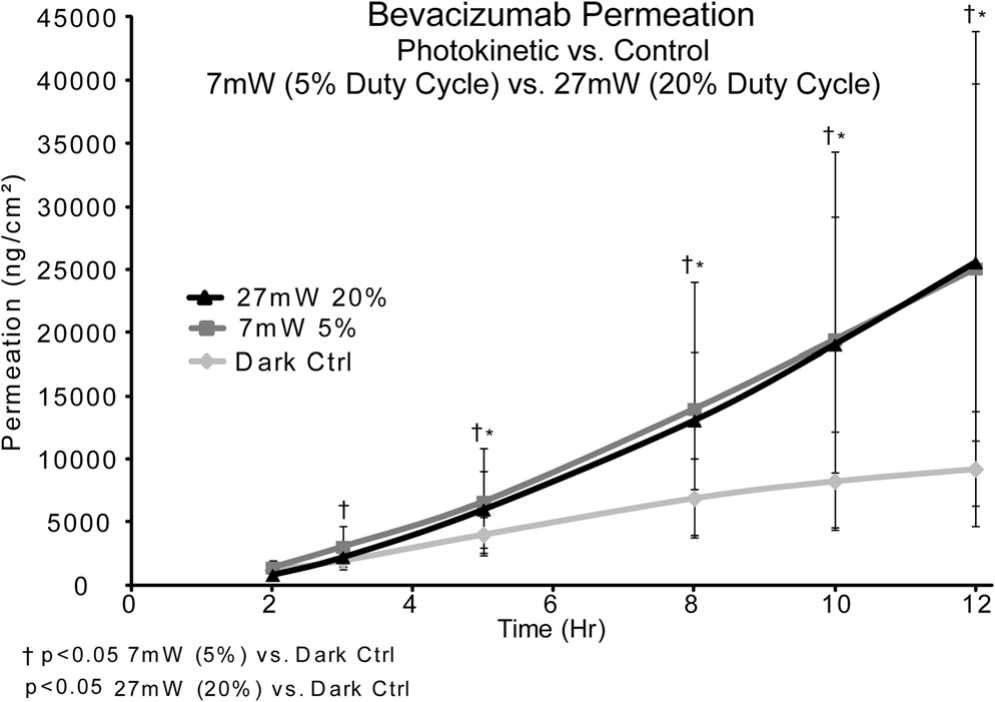
Comparison of 1 h. exposure bevacizumab, 7 mW (5%) *vs*. 27 mW (20%) duty cycle (control  n= 16, 7 mW  n= 7 and 27 mW n = 7).

Figure 5 shows the results of a study comparing the power output from the LEDs (5% *vs*. 20% duty cycle) at a wavelength of 950 nm. The two mAb permeation profiles (LED irradiated) are also compared against the permeation profile without LED irradiation (dark control). When compared, both of the LED enhanced mAb permeation profiles are about 3 times that of the dark controls. However, when comparing mAb enhanced permeation from 5% duty cycle power against 20% duty cycle power, both conditions led to nearly identical results in the irradiated sclera. Judging from these results, there is no significant difference in mAb transscleral permeation when using NIR light at 5% or 20% duty cycle power.

### ELISA ***vs***. SE-HPLC

**Fig. 6:**
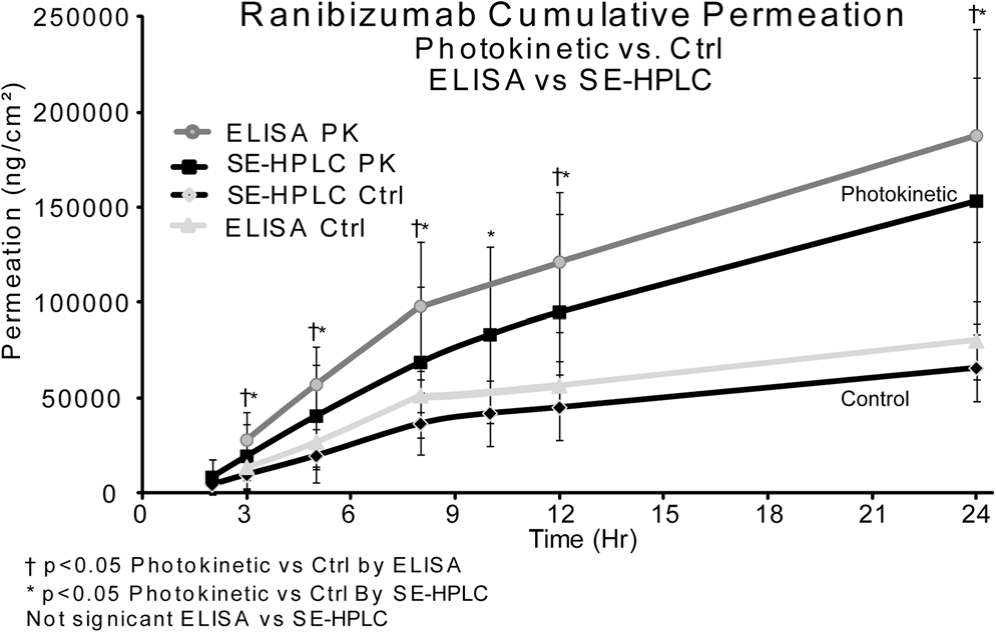
ELISA *vs*. HPLC values ≤9% difference, no significant difference (control n = 8 and 950nm n = 8). Same time point samples handled the same and split in two for analysis. HPLC samples did not require dilution for analysis. ELISA samples required several step dilutions of 100 to 300 times (F14) to bring samples into ELISA kit range of 1-100 ng/ml. All ELISA samples plated in duplicate on same plate.

Ranibizumab by ELISA and SE-HPLC correlate closely. The minor differences, seen in Fig. 6, are probably due to dilution factors and inherent error associated with ELISA. There are values of about ≤9% difference; therefore there is no significant difference in ELISA analysis *vs* SE-HPLC analysis.

### Ranibizumab with NIR 950 nm

**Fig. 7:**
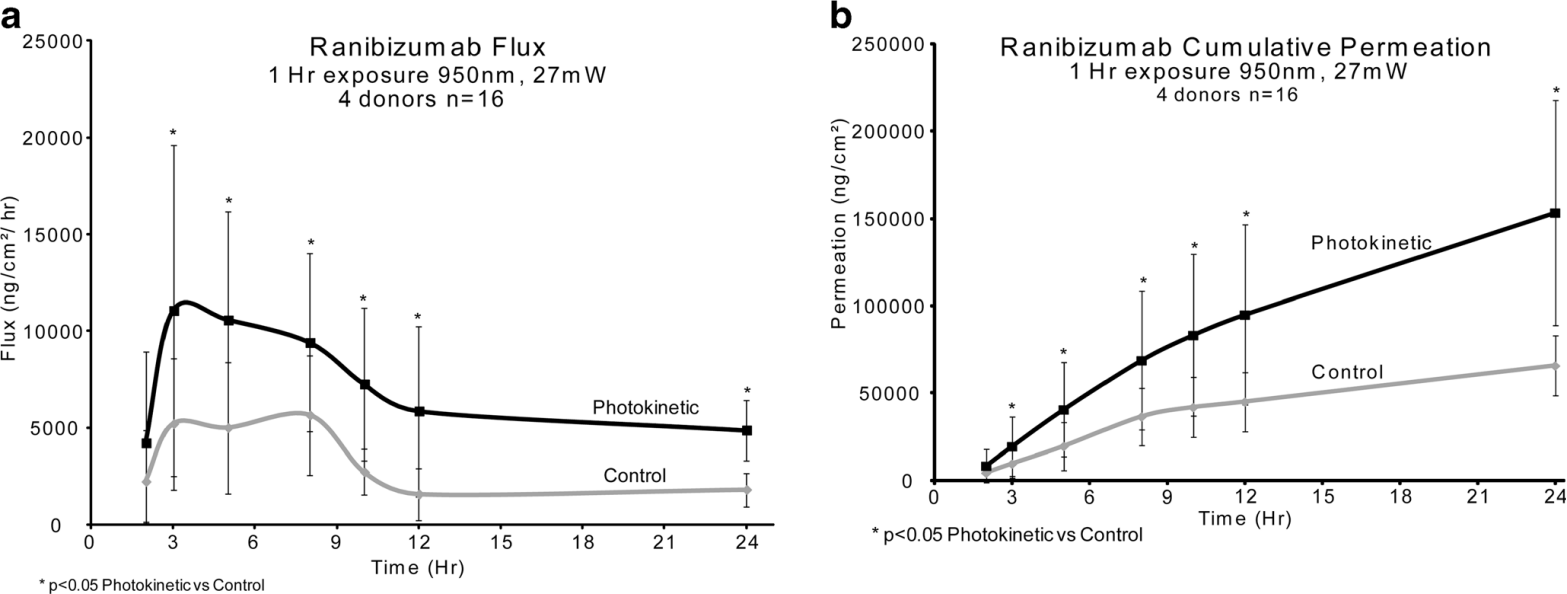
Ranibizumab with IR 950 nm (control n = 16 and 950nm n = 16). Control *vs* 950 nm/1000cps/20% duty cycle. Flux results through sclera (**a**) and cumulative permeation results (**b**) when diluted with F14.

With the one hour application of ranibizumab and concurrent 950 nm NIR irradiation, shown in Fig. 7, the flux values are twice as much as the control flux values (Fig. 7a). This overall 2X increase of ranibizumab flux, when compared to control values is consistent over the 24 h time period. The cumulative amount of drug delivered, when compared to control values, is about 2.3X after 24 h (Fig. 7b).

### Aflibercept with NIR 950 nm

**Fig. 8:**
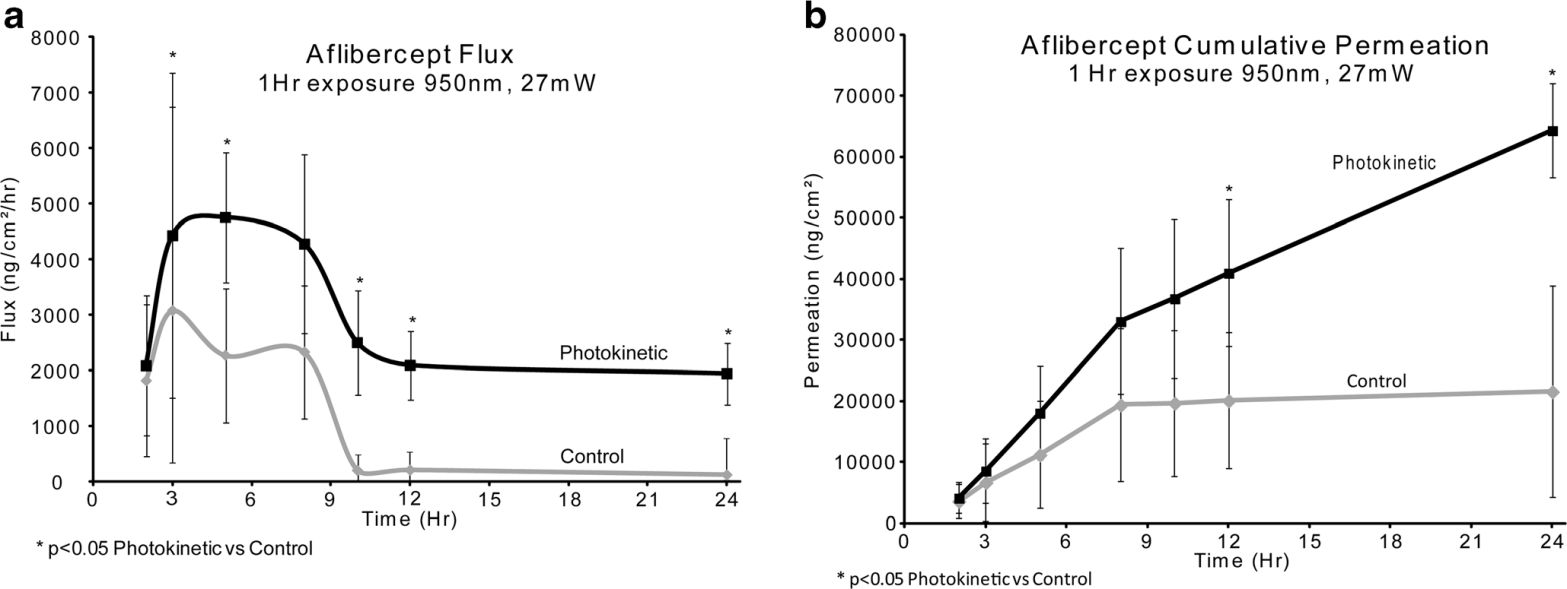
Aflibercept with IR 950 nm. (control n = 4 and 950nm n = 4). Control *vs* 950 nm/1000cps/20% duty cycle. Flux results through sclera (**a**) and cumulative permeation results (**b**) when diluted with F14.

With the one hour application of aflibercept and concurrent 950 nm NIR irradiation, the flux values are twice as much as the control flux values, as shown in Fig. 8a. This overall 2-3X increase of aflibercept flux, when compared to control values is consistent over the 24 h time period. The cumulative amount of drug delivered, shown in Fig. 8b, is about 3X, when compared to controls after 24 h.

### Comparison of Passive Transscleral Permeation

**Fig. 9:**
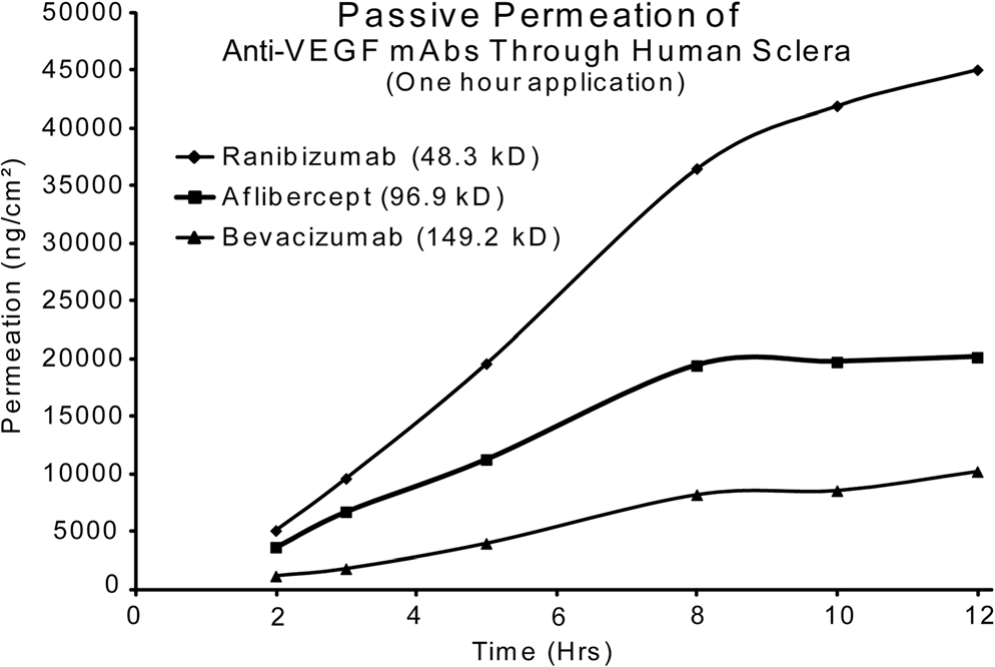
Comparison of passive transscleral permeation of ranibizumab, aflibercept and bevacizumab, through human sclera - when formulated with F14 and a residence time of 1 h (bevacizumab n = 16, ranibizumab n = 16 and aflibercept n = 4).

Earlier experiments reveal a passive, transscleral permeation due only to the mAbs being formulated in F14. Data from the ranibizumab, aflibercept and bevacizumab permeation controls (shown in Fig. 9) was compared in order to illustrate the enhancement without any light irradiation. The rate of permeation is directly proportional to the molecular weight of the mAb.

## Discussion

We became interested in the stability of dilute mAb solutions when we were developing and validating methods for mAb photokinetic transscleral permeation. mAbs are usually formulated in concentrations of 1 mg/ml or higher for intraocular injections. In general, most pharmaceutical antibody preparations contain 10-150 mg antibody/ml providing long term storage stability. Due to our planned work with Franz cells and tissue permeation, we needed to develop methods for working with dilute solutions of mAbs.

To briefly explain, the Franz Cell chamber (Fig. 1) is an *in vitro* tissue permeation assay frequently used in transmembrane feasibility and formulation development. The Franz Cell apparatus consists of two primary chambers separated by a membrane. The test product is applied to the membrane via the top chamber (donor). The bottom chamber (receiver) contains fluid from which samples are taken at regular intervals for analysis. This testing determines the amount of active compound that has permeated the membrane at each point in time.

Published tissue permeation (Franz cell) studies typically use PBS as the recipient media at 37°C. Franz cell studies start with a recipient media at 0 concentration. As the permeation experiment progresses, the concentration of the drug in the receiver chamber increases. However, at early points in the experiment, the drug is still a very dilute solution.

Body temperature and low drug concentrations in PBS (or other common media) contribute greatly to mAb aggregation, causing reduced measured concentrations and decreased VEGF binding activity. Therefore, a need arose to quickly characterize mAb dilute solutions and develop a formulation that would allow for the stable and accurate handling of these solutions in our permeation experiments.

Based upon the manufacturer’s formulation, excipient substitutions were screened with dilutions of standard concentrations of ranibizumab. An excipient formulation, that we here designate as Formula 14 (F14), developed in our lab, was chosen, which preserves and stabilizes antibody products such as bevacizumab, ranibizumab and aflibercept. Standard dilutions of bevacizumab, ranibizumab and aflibercept were prepared in PBS, manufacture’s formulation, and the new formulation. These were analyzed by HPLC and ELISA. (23) The novelty of both using and correlating the two analytical methods together ensured that the permeated drug through the scleral tissue was unchanged mAb and that the mAb was still biologically active.

Non-invasive transscleral drug delivery has been accomplished through iontophoresis, (26–28) sonophoresis (29–32) and photokinetics. (19–21) Eljarrat-Binstock *et al*. described the iontophoretic transscleral delivery of methotrexate in rabbit eyes. (33) Pescina, *et al*., used iontophoresis to deliver transscleral delivery of bevacizumab and dexamethasone in preclinical models. (27) Pulsed high-intensity focused ultrasound has been also studied recently in order to facilitate drug delivery across the sclera noninvasively. Cheung *et al*. used ultrasound (sonophoresis) to enhance the intrascleral penetration of protein, increasing the diffusivity by 1.6-folds while causing no damage to the retinal tissues. (32) Suen *et al*. also found that low-frequency ultrasound significantly enhanced the penetration of macromolecules via transscleral route. (29)

Photokinetic ocular drug delivery (PODD) describes drug permeation enhancement through the use of pulsed infrared light for ophthalmic applications. Briefly, it is hypothesized that if a drug molecule in a pharmacologically acceptable formulation is placed on the surface of the sclera/cornea and cyclically illuminated with a selected wavelength of NIR light at a selected pulse rate, that drug molecule and/or tissue would absorb the light, which would result in molecular bond vibrations. This cyclic molecular bond stretching and relaxing would in turn cause a molecular kinetic motion. The resulting cyclic physical shape change of the molecule and/or tissue may cause gross movement and result in the facilitated diffusion of the molecule into and through the sclera/cornea membrane. (21,24)

Bevacizumab was tested to verify that NIR light was not damaging to the protein. Both ELISA and SE-HPLC methods (shown in Fig. 2) were used for analysis. The bevacizumab concentrations obtained for the control (no NIR light) and light exposed (NIR light) groups, as analyzed by SE-HPLC, were both about the same quantity at 1 and 5 h, respectively. The bevacizumab concentrations obtained for the one hour control and light exposed group were also about the same quantity, using ELISA as the analytical method. At 5 h, both the control and the light exposed groups showed a slight increase in concentration, when analyzed by ELISA. This slight increase in concentration may be attributed to the inherent error in ELISA methodology and/or standard curve dilution or interpolation errors. For example, Barregard *et al*., studying urinary 8-oxo-7,8-dihydro-2′-deoxyguanosine (8-oxodG); a widely used biomarker of oxidative stress, found that chromatographic assays showed high agreement across urines from different subjects, whereas ELISAs showed far more inter-laboratory variation and generally overestimated levels, compared to the chromatographic assays. (34) Additionally, errors in plate manufacturing, handling, loading, or mistakes in the usage of reagents are all common causes of well variance in ELISA. (35)

In our *in vitro* studies, we did not vary the length of time for the application of the antibody solution or the length of time of NIR irradiation. These parameters were held constant at 60 min. This 60 min treatment protocol is central to our envisioned mode of therapy. We found that applying pulsed NIR light at 950 nm increased the amount of drug to permeate through scleral tissue (Figs. 3, 4, 5, 6, 7, and 8). Figure 9 shows the amount of passive transscleral permeation of each mAb as derived from the control values of the previous figures.

We investigated several different LEDs, with variable attainable light powers for each, as previously described. The distance of the LED to the tissue surface was constant, however the irradiated power from each test condition varied. During the NIR irradiation of the mAb solution study, we used LEDs positioned 3 cm above the surface of the drug solution (950 nm). The LED driver currents were adjusted to provide an irradiation power of 12 mW ± 1 mW at 3 cm away from the LED. These solutions were continuously irradiated for 5 h, with samples taken at 1 and 5 h; the irradiated samples and the dark control samples were treated the same. The total light dosage (power x time) at 5 h exceeded all the Franz test conditions of one hour light exposure used in any tissue permeation experiment. In the Franz cell permeation experiments, the LED was positioned at 1 cm above the tissue surface and approximately 0.5 cm above the drug donor. This 1 cm distance was selected for the reason that the average vertex distance of spectacle lenses (the distance between the front of the cornea and the back surface of a corrective lens), when properly fitted, is 8–12 mm. Many refractive surgeons assume an average vertex distance of 12 to 14 mm for all patients without measuring vertex distance. The average true vertex distance has been reported to be 20.4 mm with a range of 10 to 34 mm. (36)

In our studies, pulsed NIR light was remotely directed onto aqueous mAb solutions, as well as mAb solutions applied onto the surface of scleral tissue. The NIR LEDs were electronically driven with short ON period pulses. Duty cycle or pulse width modulation specifications of 5–20% (5–20% of the total cycle time being ON) eliminates heat buildup within the LED itself. (37) Absorbed light energy transformed into heat is the primary degrading pathway for exposed tissues, cells and proteins. Direct heating, for example, from contact with the LED itself could be another source of damage. (37)

In our configuration, the LED is not in contact with the drug, drug solution or tissue surface. Therefore, the possibility of direct heat damage from the LED is not an issue. For light energy to be transformed into heat requires that the light be absorbed. Water is the primary light absorbing material at 950 nm. Our direct temperature measurements of the Franz drug donor solutions indicated minimal temperature rises of ≤1°C above the 37°C water bath. This value, being well below a destructive thermal injury temperature, indicates that the possibility of heat damage to tissues or proteins is insignificant.

Non-thermal red to NIR light is non-ionizing and is thought to be of no consequence and may actually be beneficial to ocular tissues. (38–40) We are not aware of any reports wherein pulsed non-thermal NIR light in the 950 nm range, at the conditions we describe, have caused damage to tissues or proteins.

These results demonstrate that narrow wavelength incoherent (non-laser) light from an NIR light emitting diode (LED) source can be used for drug delivery and that non-ionizing pulsed NIR light with these characteristics is not harmful to the drug molecule.

In addition to drug permeation enhancement, we also saw a drug depot effect. A drug depot effect can be defined as a body area in which a substance, e.g., a drug, can be accumulated, deposited, or stored and from which it can be distributed. We observed that although the drug solution was in contact with the sclera for a relatively short time - one hour with concurrent IR light, and then both the drug solution and light removed - there was continuous and enhanced drug diffusion of the drug over the next 23 h when compared to “no light” controls. The application of NIR light generates a photokinetic action, thereby enhancing drug absorption into the outer layers of scleral/corneal tissue, which leads to an overall 2-3X increase in drug permeation over a prolonged amount of time. In this sense, there is improved and enhanced tissue penetration, depot effect and sustained release drug action.

Intravitreal injection of anti-VEGF therapies are commonly performed every 4–8 weeks to treat retinal disease (Table II). This treatment regimen is based mainly on the patient tolerability of repeated injections, rather than maintaining optimum binding concentrations of anti-VEGF drugs to their target. Aflibercept’s competitive advantage over ranibizumab is attributed to an every-two-month intravitreal injection treatment compared to a once-a-month injection required with ranibizumab. This indicates that patients prefer less invasiveness.

**Table II:** Current clinical intravitreal injection protocols

	Ranibizumab	Aflibercept	Bevacizumab
Dosage	0.5 mg (0.05 ml, 10 mg/ml) Every 28 days	2.0 mg (0.05 ml, 40 mg/ml) Every 4–8 weeks	1.25 mg (0.05 ml, 25 mg/ml) Every 28 days
Concentration*	0.123 mg/ml	0.493 mg/ml	0.309 mg/ml

*Concentration in vitreous after injection. Assume vitreous body is about 4 ml

Comparing once-a-month intravitreal injection to a more frequent (1 or 2 times a week) noninvasive topical treatment has to take into account the therapeutic concentration requirement, not the dose administered. Injected anti-VEGF mAb concentrations, in the vitreous, have large initial peaks with long low concentration troughs. (41) The biological half-life of bevacizumab and ranibizumab is found by about day 8 after injection. By day 14, the vitreal concentration of injected mAbs is generally insignificant. More frequent dosing, every 2 weeks, has been shown to have greater efficacy. (42,43) A more effective treatment would be for more frequent dosing; however patents will generally not tolerate an every-2-week treatment regime. Non-invasive frequent topical dosing may not reach the high peak levels of an injection, but may provide a constant therapeutic level within the eye. This concept is the foundation of non-invasive ocular delivery, (44) as well as for controlled release from reservoir implants for posterior segment disease. (45)

Any topical mAb ocular therapy will require modification of the antibody carrier composition to be physiologically acceptable (pH, osmolarity and viscosity) as well as pharmacologically acceptable (excipients used to prevent antibody aggregation) for application onto the eye. Ocular compositions need to be non-irritating and provide enough resident time for ocular tissue absorption. We have previously determined that anti-VEGF antibodies undergo significant aggregation unless we modify the carrier composition. (23) Our composition provides monomeric antibody forms that can be used for tissue permeation in a clinical setting.

Our Franz cell drug donor; a topical formulation with a concentration of 2.5 mg/ml, was based on recent anti-VEGF topical therapies for corneal neovascularization (46,47), as well as prior permeation cell work (27) (see Table III). In these prior studies, existing compositions were diluted with saline and applied to the eye or eye tissue. The concentration of 2.5 mg/ml was used as a starting point for the studies to relate to prior work by others as well as provide a method to suspend antibodies in our anti-aggregation composition.

**Table III:** Topical anti-VEGF agent concentrations of various studies

Anti-VEGF Agent	Concentration	Dose	Model	Author
Aflibercept Aflibercept Bevacizumab	4 mg/ml 0.4 mg/ml 2.5 mg/ml	Not specified	Rabbit	Park 2015 (46)
Bevacizumab	2.5 mg/ml	2X/day	Human	Krizova 2014 (47)
Bevacizumab	10 mg/ml	2X/day	Rat	Ozdemir 2014 (48)
Bevacizumab	6 mg/ml 25 mg/ml	2X/day	Rabbit	Kadar 2014 (49)
Ranibizumab	10 mg/ml	4X/day	Human	Ferrari 2013 (50)
FITC labeled Bevacizumab	2.67 mg/ml	0.534 mg (0.2 ml, 2.67 mg/ml)	Franz cell	Pescina 2010 (27)
Bevacizumab	12.5 mg/ml	3X/day	Rabbit	Ahmed 2009 (51)
Bevacizumab	10 mg/ml	2X/day 4X/day	Human	Dastjerdi 2009 (52)
Bevacizumab	5 mg/ml	Eye drops, 5X/day	Human	Koenig 2009 (53)
Bevacizumab	5 mg/ml	5X/day	Human	Bock 2008 (54)
Bevacizumab	12.5/ml	2X/day	Human	Kim 2008 (55)
Bevacizumab	10 mg/ml	4X/day	Human	DeStafeno 2007 (56)

The *in vitro* flux data provides information for the *in vivo* situation. However, this information cannot fully simulate and account for the actual limited drug residence time on the tissue surface, due to tear washout time, or the restrictions from multiple tissue layers under the sclera. On the other hand, the *in vitro* conditions may not include increases in drug flux due to increased distribution from drug donor/tissue contact such as eye blinking or the increased available permeation area found when the entire anterior eye surface is considered. There also remains an added possible drug reservoir effect and prolonged drug elution from the conjunctiva into the eye.

As shown in our permeation model results, we have attained clinically relevant drug fluxes with a non-stirred, one-hour application of 0.4 ml of a 2.5 mg/ml solution (1 mg dose) related to one square cm of sclera permeation area. We used sclera sections from human eyes immediately adjacent to the cornea representing a clinically relevant tissue treatment location and tissue thickness. In the case of ranibizumab, which is clinically dosed at 0.5 mg /injection, the monthly dose is 500,000 ng. In our experiment, the dose given was 1,000,000 ng. Our transscleral results were 60,000 ng/24 h passively and 150,000 ng/24 h using 60 min of NIR. If a one hour NIR treatment was given once a week for 4 weeks, this would equal 600,000 ng which is comparable to the injection dose.

Drug permeation into and through tissues is time, concentration and carrier dependent. The Franz cell model predicts *in vivo* permeation and drug fluxes under specified conditions. The translation from predicted fluxes with the permeation cell model into actual achievable *in vivo* drug fluxes can only be done by direct *in vivo* models. We are currently planning pre-clinical studies to evaluate the mAb formulation and photokinetic drug delivery enhancement.

## Conclusion

We first developed a novel pharmacologically acceptable formulation that prevented antibody aggregation and then combined it, *in vitro*, with our enhanced, near infrared (NIR) light-based transscleral drug delivery system.

*In vitro* human sclera permeation methods were adapted to include concurrent irradiation with non-thermal, pulsed 950 nm NIR light from an LED source. We observed that although the drug solution was in contact with the sclera for a relatively short time - one hour with concurrent NIR light, and then both the drug solution and light removed - there was continuous and enhanced drug diffusion as well as drug elution from the sclera over the next 23 h.

Pulsed, non-thermal NIR irradiation is non-visible light, which is more preferred; being less annoying to a patient, harmless to eye tissues. Exposure to pulsed, non-thermal NIR light has no degrading effect on mAbs, when comprised with our formula. The optimal LED wavelength for enhanced permeation was 950 nm. The duty cycle (ON time: total cycle time) of 5% *vs* 20% showed no difference in enhanced permeation. The topical formulation, combined with pulsed NIR light irradiation, significantly improved scleral permeation of three anti-VEGF agents compared to control conditions.

The combination of a non-aggregating antibody formulation and pulsed NIR irradiation, provided an average enhancement factor of 3.0 (range is 2.5–3.5X), when compared to passive permeation. This combination of stabilized antibody with light irradiation provides clinically relevant drug amounts to be delivered into the eye using a one-hour treatment avoiding needle injection. A one-hour non-invasive, drug/NIR treatment, given once a week for 4 weeks, would equal the same dose given by a monthly injection. Our model serves as a good case for the importance of topical compositions preventing antibody aggregation as well as an indication that topical antibody delivery for posterior segment treatment is possible and likely.

### ACKNOWLEDGMENTS AND DISCLOSURES

The authors wish to acknowledge and thank Genentech, South San Francisco CA, for the gift of Lucentis and Regeneron for the gift of IAI Eylea. The authors Steven A. Giannos and Edward R. Kraft own significant interest in the IP of the technology described. The authors Zhen-Yang Zhao, Kevin H. Merkley and Jiyang Cai have no competing interests.

## ABBREVIATIONS

AMD: Age related macular degeneration
cps: Cycles per second
ELISA: Enzyme-linked immunosorbent assay
IV: Intravenous
LED: Light emitting diode
mAb: Monoclonal antibody
NIR: Near infrared
PBS: Phosphate buffered saline
PODD: Photokinetic ocular drug delivery
SE-HPLC: Size exclusion high-performance liquid chromatography
VEGF: Vascular endothelial growth factor
